# Computed tomography-guided cryoablation for adrenal metastases: local control and survival

**DOI:** 10.1097/MD.0000000000013885

**Published:** 2018-12-21

**Authors:** Wei Zhang, Li-Jun Sun, Jian Xu, Yu-Fei Fu, Zhi-Xiang Zhuang

**Affiliations:** a Department of Oncology, Second Affiliated Hospital of Suzhou University, Suzhou; b Department of Infectious Disease; c Department of Radiology, Xijing Hospital, Xi’an; d Department of Radiology, Xuzhou Central Hospital, Xuzhou, China.

**Keywords:** adrenal, computed tomography, cryoablation, metastases

## Abstract

To evaluate the feasibility, local control, and survival after computed tomography (CT)-guided cryoablation for adrenal metastases.

This study included 31 consecutive patients with adrenal metastases who were treated by CT-guided cryoablation in our center from July 2011 to October 2017. The technical success rate, local progression rate, local progression-free survival (LPFS), systemic progression-free survival (SPFS), and overall survival were assessed. The predictors of survival were determined using univariate and multivariate Cox regression analyses.

The primary and secondary technical success rates were 90.3% and 100%, respectively. None of the patients experienced a hypertensive crisis. The local progression rate during follow-up was 19.4%. Systemic progression was found in 9 patients. The cumulative 1-, 3-, and 5-year LPFS rates were 80.6%, 37.8%, and 18.4%, respectively. The cumulative 1-, 3-, and 5-year SPFS rates were 77.4%, 31.9%, and 14.6%, respectively. The cumulative 1-, 3-, and 5-year overall survival rates were 83.9%, 45.0%, and 30.0%, respectively. The existence of an extra-adrenal tumor was a significant independent predictor of worse overall survival (*P* = .012). The mean overall survival durations were significantly different between patients with and without an extra-adrenal tumor (16.6 ± 2.4 vs 50.9 ± 4.5 months, *P* <.001).

Our findings support that CT-guided cryoablation is a safe and effective method for controlling adrenal metastases and imply that this approach may improve the survival of patients with adrenal metastases.

## 1 Introduction

The adrenal glands are a common site for metastases, which are usually from lung cancer, renal cancer, colorectal cancer, hepatocellular carcinoma (HCC), and malignant melanoma.^[1–3]^ Autopsy studies have identified adrenal metastases in up to 27% of patients with known malignancies.^[4,5]^ Although no randomized controlled trials have demonstrated the benefits of local treatment for adrenal metastases, several investigators have emphasized the utility of surgical resection for improving the survival of selected patients with isolated adrenal metastases.^[6–10]^ However, adrenalectomy is not always feasible owing to patient comorbidities and may be associated with prolonged hospital admissions.^[1–3]^ In addition, the adrenal glands are retroperitoneal organs, which usually require complex surgical procedures. Hence, other methods of controlling and treating adrenal metastases are needed.

At present, computed tomography (CT)-guided radiofrequency and microwave ablation is used to treat adrenal metastases,^[1,2]^ and local recurrence-free survival rates of 70.5% to 82% at 1 year have been reported.^[1,2]^ Recently, CT-guided cryoablation has gained increased attention as an approach for managing several malignant tumors due to its advantageous features of a visible treatment zone, reduced pain, and improved healing.^[11,12]^ However, the clinical effectiveness of cryoablation for adrenal metastases remains unclear.

Therefore, the purpose of the present study is to evaluate the feasibility, local control, and survival rates after using CT-guided cryoablation as a treatment for adrenal metastases.

## 2 Materials and methods

This single-center retrospective study was approved by our Institutional Review Board. The requirement of written informed consent was waived.

### 2.1 Study design

For this study, we recruited consecutive patients with adrenal metastases who were treated with CT-guided cryoablation in our center from July 2011 to October 2017. The inclusion criteria were as follows:

(a) patients who were not considered surgical candidates or who declined to undergo surgical intervention;
(b) tumor size ≤5 cm;
(c) no or controlled extra-adrenal tumors; and
(d) a life expectancy ≥3 months.

The exclusion criteria were as follows:

(a) patients with adrenal vein invasion; and
(b) significant dysfunction of blood coagulation, active infection, and/or active bleeding.

### 2.2 Diagnosis

Adrenal metastasis was diagnosed based on each patient's history, abdominal CT/magnetic resonance imaging (MRI) findings, and biopsy results. All patients also underwent chest, pelvic CT, brain MRI, and bone emission CT to detect the presence/absence of extra-adrenal tumor(s).

### 2.3 Cryoablation

Each patient was placed in the prone position. All procedures were performed by 3 interventional radiologists under the guidance of a spiral CT system (PQ6000, Philips, Amsterdam, Netherlands).

Argon-helium cryoablation was performed using a cryoablation system (Cryo-hit, Galil Medical, Israel) that employed argon/helium gases and as many as 25 cryoprobes (1.47 mm in diameter). The freezing area of 1 cryoprobe was 1.5 × 3.5 cm. Before cryoablation, each patient underwent CT scanning to confirm the location, size, and extent of the tumor. The number of cryoprobes used was selected based on the size of the tumor. The puncture sites and distribution of the cryoprobes were determined by the location, shape, and surrounding structure of the tumor. The edge of the freezing area was 0°C, but temperatures less than -40°C are needed to kill the cells; therefore, the freezing area was set 0.5 to 1 cm beyond the tumor edge.^[13]^ The interval between 2 cryoprobes was ≤1.5 cm.^[13]^

After confirming the puncture sites, each patient was administrated local anesthesia with 5 mL of 2% lidocaine. Once the cryoprobe had been placed, the tumor was treated with 2 freeze-thaw cycles (10-min freeze phase and 3-min thaw phase for each cycle). The rapid expansion of argon gas in a sealed cryoprobe with a distal uninsulated portion resulted in rapid freezing of the tumor tissue, and cryoprobe tip temperatures reached a nadir of approximately -140°C within a few seconds. Thawing was accomplished by replacing the argon gas with helium gas. After the treatment, all patients underwent CT scanning immediately to confirm the dimensions of the generated ice balls.

### 2.4 Postoperative management and follow-up

Following cryoablation, patients were carefully observed for 30 minutes, and then returned to the ward if they were not experiencing any discomfort. Patients’ vital signs were closely monitored for the first 6 hours after treatment. Each patient was managed with appropriate anti-inflammatory and hemostasis treatments for 3 to 5 days after cryoablation.

The follow-up period ended at the time of death or at the last visit of the patient until June 2018. Routine physical examinations and laboratory tests, including assessments of blood cell counts, adrenal hormone levels, and tumor markers based on primary tumor histologic findings, were performed every month. Chest, abdominal, and pelvic CT scans with and without contrast, material enhancement was obtained 1, 3, 6, and 12 months, and then every 6 months, after cryoablation.

### 2.5 Definitions

Primary technical success was defined as completion of cryoablation with a planned treatment protocol and no visible tumor enhancement on the initial contrast-enhanced CT or MR images obtained 2 to 5 days after treatment, as the ablation zone in the surrounding fat tissue was difficult to detect on CT or MR images.^[1]^ When tumor enhancement remained, the treatment was repeated 1 week later. Secondary technical success was defined as the achievement of technical success after the first and second cryoablation treatments for the residual tumor.^[1]^

Local tumor progression was defined as the development of a new enhancing adrenal tumor during the follow-up period after technical success of cryoablation.^[2]^ Systemic progression included local tumor progression, the development of new metastases, or progression of the primary tumor.

### 2.6 Statistical analysis

All statistical analyses were performed using SPSS 16.0 (SPSS Inc., Chicago, IL). Continuous variables are presented as the mean or median. Continuous variables and numeric data between patients with and without extra-adrenal tumor were compared by *t* tests and χ^2^ tests/Fisher exact probability tests respectively. Survival times were calculated using Kaplan–Meier curves and survival time between patients with and without extra-adrenal tumor were compared by log-rank tests. The predictors of survival were determined using univariate and multivariate Cox regression analyses. The covariates incorporated into the multivariate analysis were the variables with *P* values <.1 in the univariate analyses. Statistical significance was set at *P* <.05.

## 3 Results

### 3.1 Patients

A total of 31 patients with adrenal metastases were treated by cryoablation. All patients had 1 adrenal tumor. The baseline data of the 31 patients are provided in Table 1. Among the 31 patients, 20 patients previously underwent surgical resection for renal cancer (n = 12), lung cancer (n = 6), and colon cancer (n = 2). No patient underwent adrenal resection before cryoablation.

**Table 1:**
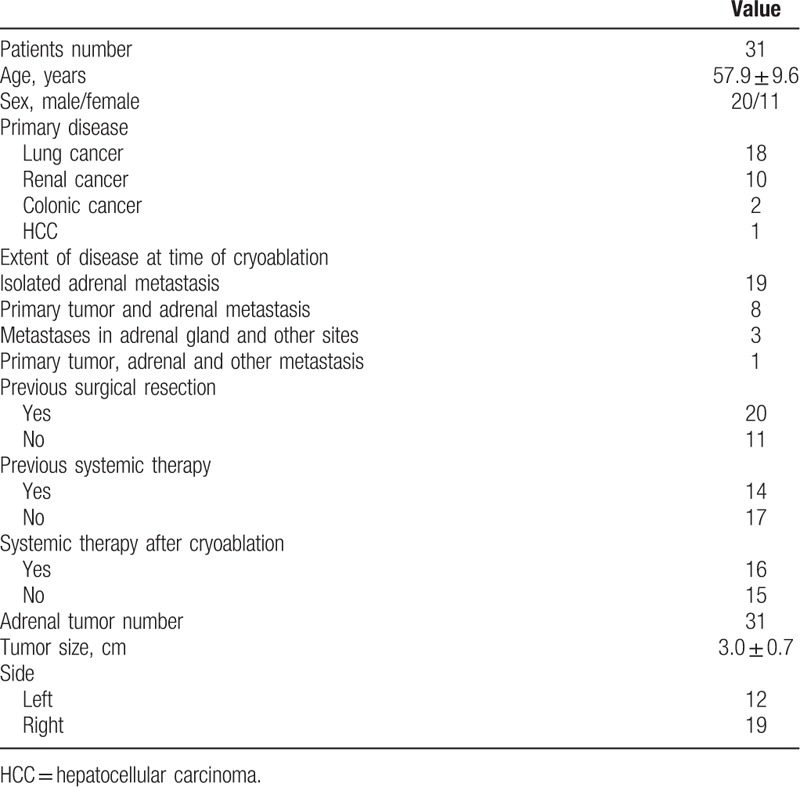
Baseline data of the 31 patients.

One patient previously underwent transcatheter arterial chemoembolization for HCC. Thirteen patients previously underwent systemic chemotherapy. Among the 14 patients who were treated by chemotherapy before adrenal cryoablation, 11 patients developed adrenal metastases during the chemotherapy and 3 adrenal metastases resisted the chemotherapy.

### 3.2 Technical success

**Figure 1:**
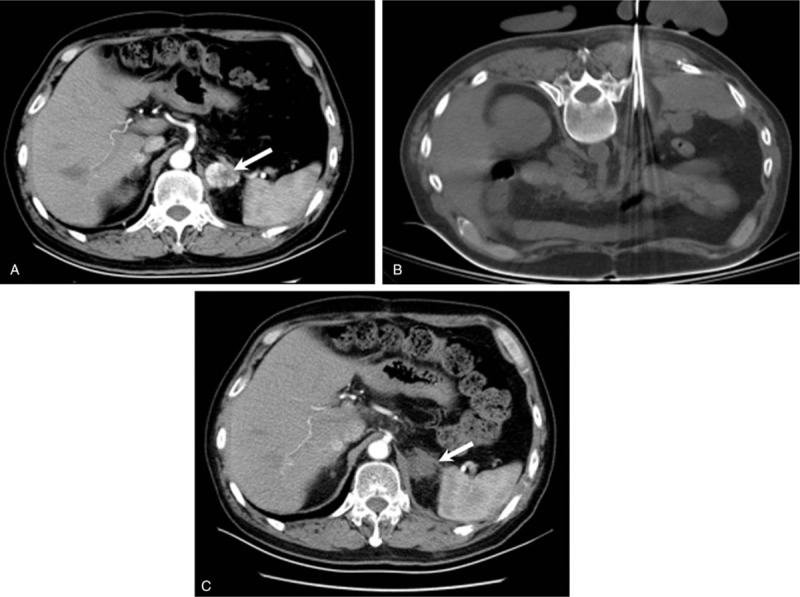
A 59-year-old man who developed left adrenal metastasis after the resection of renal clear cell carcinoma. (A) Contrast-enhanced CT scan showing a tumor (arrow) in the left adrenal gland. (B) Procedures for cryoablation. (C) Contrast-enhanced CT scan showing that the tumor enhancement is no longer visible (arrow). CT = computed tomography.

The primary technical success rate was 90.3% (28/31 patients, Fig. 1). Residual tumor enhancement remained in 3 patients, all of whom underwent repeated cryoablation. The secondary technical success rate was 100%.

### 3.3 Complications

Nine patients experienced mild blood pressure increases during the cryoablation procedure, but the blood pressure in these patients quickly returned to normal after the administration of alpha blockers. None of the patients experienced a hypertensive crisis during the procedure. Six patients experienced back pain after cryoablation, but the back pain gradually dissipated in all patients.

### 3.4 Local progression

**Figure 2:**
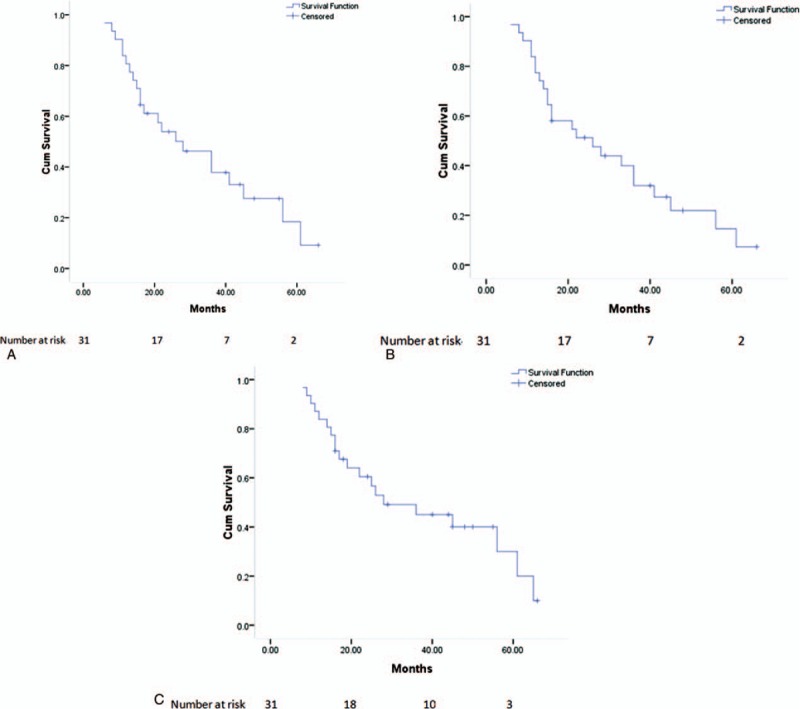
Kaplan–Meier curves of local progression-free survival (A), systemic progression-free survival (B), and overall survival (C).

During a follow-up period of 8 to 66 months (mean: 30.5 ± 18.2 months), 16 patients underwent systemic therapy after cryoablation. Among these 16 patients, 6 patients had an extra-adrenal tumor. Six (19.4%) of the 31 patients experienced local progression within 6 to 41 months (median: 17 months). Five patients underwent repeated cryoablation, and 1 patient refused to receive further treatment. The mean local progression-free survival (LPFS) duration was 32.7 ± 3.9 months (95% confidence interval [CI]: 25.1–40.3). The cumulative 1-, 3-, and 5-year LPFS rates were 80.6%, 37.8%, and 18.4%, respectively (Fig. 2A).

### 3.5 Systemic progression

Systemic progression was found in 9 patients, including local adrenal progression (n = 6), recurrence of the primary tumor (renal cancer: n = 1; colon cancer: n = 1), and development of a new metastasis (n = 1). The mean systemic progression-free survival (SPFS) duration was 30.4 ± 3.6 months (95% CI: 23.3–37.6). The cumulative 1-, 3-, and 5-year SPFS rates were 77.4%, 31.9%, and 14.6%, respectively (Fig. 2B).

### 3.6 Overall survival

Twenty patients died during the follow-up period. The causes of death included tumor progression (n = 16), lung infection (n = 3), and liver failure (n = 1). The mean overall survival duration was 37.1 ± 4.2 months (95% CI: 29.0–45.3). The cumulative 1-, 3-, and 5-year survival rates were 83.9%, 45.0%, and 30.0%, respectively (Fig. 2C).

The univariate and multivariate Cox regression analyses revealed that the existence of an extra-adrenal tumor (hazard ratio: 6.5, 95% CI: 1.513–27.784, *P* =.012) was a significant independent predictor of worse overall survival (Table 2). The comparison of baseline data between patients with and without extra-adrenal tumor is provided in Table 3. The mean overall survival durations were significantly different between patients with and without an extra-adrenal tumor (16.6 ± 2.4 vs 50.9 ± 4.5 months, *P* <.001).

**Table 2:**
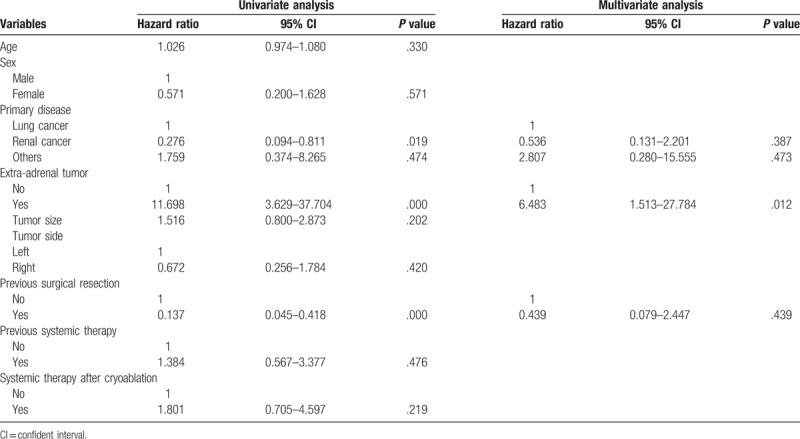
Univariate and multivariate Cox analyses of overall survival.

**Table 3:**
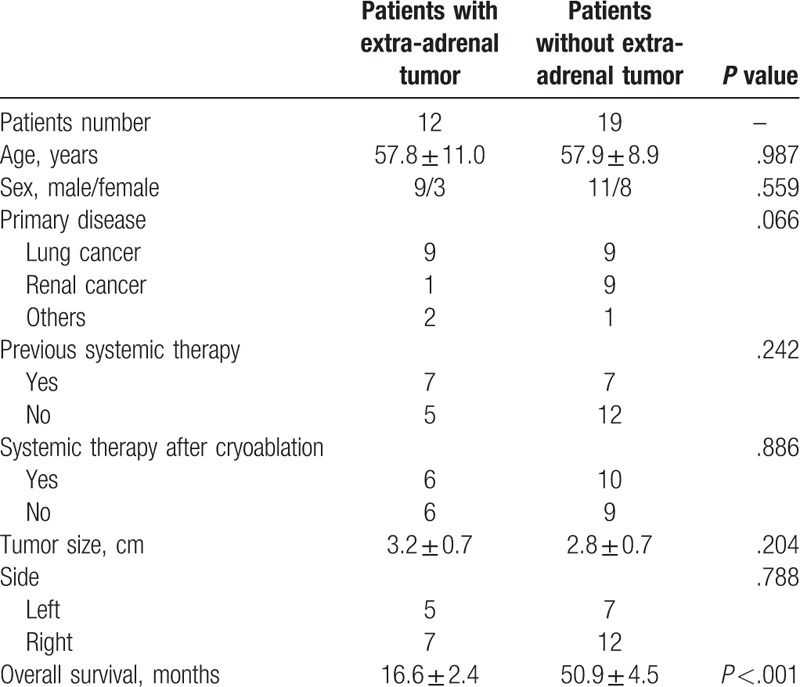
Comparison of details between patients with and without extra-adrenal tumor.

## 4 Discussion

The results of the present study show that cryoablation is a safe and effective treatment method for adrenal metastases ≤5 cm. The primary and secondary technical successful rates were 90.3% and 100%, respectively. These rates are comparable to the rates reported for other ablation methods (e.g., radiofrequency and microwave ablation).^[1,2,14,15]^ The primary complete ablation rate of 90.3% (28/31) indicated that cryoablation has a strong ability of damaging the tumor tissue. Although some patients might had residual tumor after the first ablation, repeat ablation could effectively provide a supplementary treatment.

Local control rate was an important endpoint in this study. Six (19.4%) of the 31 patients in our study experienced local recurrence of the adrenal tumor. This rate is comparable to the rates reported in previous studies that used microwave (15.2%) and radiofrequency (23%) ablation.^[1,16]^ Here, the median time to local progression was 17 months, which was within the range reported in prior studies that employed microwave (24 months) and radiofrequency (8.6 months) ablation.^[1,16]^ These results may indicate that cryoablation has a similar effect in local control of adrenal metastases to the thermal ablation methods. In addition, our local control rate (80.6%) was almost equal to that of adrenalectomy (77%–83%).^[3,4]^

Overall survival rates at 1-, 3-, and 5 years of 83.9%, 45.0%, and 30.0% in this study are comparable to those reported in previous studies that used other ablation approaches to treat adrenal metastases.^[1,2]^ Univariate and multivariate Cox regression analyses identified the existence of an extra-adrenal tumor as an independent predictor of worse overall survival. Accordingly, we found a significant difference in the overall survival duration between patients with and without an extra-adrenal tumor. Similarly, a prior study that used radiofrequency ablation to manage unresectable adrenal metastases demonstrated that the existence of extra-adrenal tumors (*P* = .005) and an age ≥65 years (*P* = .04) were significant predictors of a poor prognosis.^[1]^ The absence of extra-adrenal tumors was also revealed as an important prognostic factor in a previous report that employed adrenalectomy.^[8]^ Probably because the disease stage is higher even after cryoablation in patients with extra-adrenal tumors, the prognosis of these patients is not good. Frenk et al.^[2]^ found that metastasis from lung cancer was a predictor of worse overall survival. However, this result was obtained only from the univariate analysis. The other studies also did not reveal that type of disease was associated with survival.^[1,8]^

Chemotherapy or radiotherapy has also been used to manage unresectable adrenal metastases related to different primary tumors.^[1,2]^ However, 11 patients developed adrenal metastases during the chemotherapy and 3 adrenal metastases resisted the chemotherapy in our study. It seems that adrenal cryoablation could be used as a salvage treatment after chemotherapy.

Three of our patients experienced extra-adrenal progression after cryoablation. Given that cryoablation is a local treatment protocol, performing follow-up evaluations with the appropriate imaging protocol is critical for identifying extra-adrenal progression and ensuring that the proper treatment is administered in a timely manner.^[11,12]^

Previously, hypertensive crisis was reported as a common major complication of adrenal ablation.^[2,16,17]^ However, none of the patients in our study experienced a hypertensive crisis. This result may be attributed to the use of cryoablation, as the prior studies used thermal ablation (radiofrequency and microwave ablation), which is more likely to induce a hypertensive crisis.^[2,16,17]^ A hypertensive crisis is particularly likely to occur with microwave ablation, because it elevates the temperature more rapidly than does radiofrequency ablation.^[17]^ Given that none of our patients developed a hypertensive crisis, this suggests that cryoablation may be a safer treatment option than thermal ablation.

Several limitations of this study should be noted. First, the retrospective design of the study may have led to some selection bias. Second, the limited sample size from a single-center makes it difficult to make definitive conclusions regarding the clinical effectiveness of the treatment and long-term outcomes of the patients. Further multiple-center, randomized controlled trials should be performed to address these issues. Third, the inclusion of multiple primary cancer types likely also caused selection bias. Although the univariate and multivariate Cox regression analyses indicated that cancer type was not associated with overall survival, research that focuses on a particular disease may provide more information about the exact contributing factors. Finally, although cryoablation resulted in excellent local control of the adrenal tumors, it produced limited systemic control of the disease. Hence, an appropriate systemic treatment protocol should be utilized after cryoablation.

In conclusion, the findings of the present study support that cryoablation is a safe and effective method of controlling adrenal metastases and suggest that it may improve the survival of patients with adrenal metastases.

## Author contributions

**Data curation:** Li-Jun Sun.

**Formal analysis:** Jian Xu.

**Methodology:** Li-Jun Sun, Jian Xu.

**Project administration:** Yu-Fei Fu.

**Supervision:** Zhi-Xiang Zhuang.

**Writing – original draft:** Wei Zhang.

**Writing – review & editing:** Yu-Fei Fu, Zhi-Xiang Zhuang.
